# The clinical outcome of pembrolizumab for patients with recurrent or metastatic squamous cell carcinoma of the head and neck: a single center, real world study in China

**DOI:** 10.3389/fonc.2024.1360657

**Published:** 2024-02-19

**Authors:** Zongyu Fan, Rongrong Hui, Houyu Ju, Yunteng Wu, Xuhui Ma, Hao Song, Yang Liu, Mengyu Rui, Xinrong Geng, Minqi Zhao, Yingye Xin, Dongliang Wei, Guoxin Ren

**Affiliations:** ^1^ Department of Oral Maxillofacial‐Head and Neck Oncology, Shanghai Ninth People’s Hospital, Shanghai Jiao Tong University School of Medicine, Shanghai, China; ^2^ Shanghai Key Laboratory of Stomatology & Shanghai Research Institute of Stomatology, National Clinical Research Center of Stomatology, Shanghai, China; ^3^ National Clinical Research Center of Stomatology, Shanghai, China; ^4^ School of Stomatology, Weifang Medical University, Weifang, China; ^5^ School of Health Science and Engineering, University of Shanghai for Science and Technology, Shanghai, China; ^6^ Department of Orthopedic Oncology, Changzheng Hospital, Second Military Medical University, Shanghai, China

**Keywords:** recurrent or metastatic squamous cell carcinoma of the head and neck, immunotherapy, pembrolizumab, cetuximab (c225), immunotherapy-related adverse events

## Abstract

**Background:**

The KEYNOTE-048 and KEYNOTE-040 study have demonstrated the efficacy of pembrolizumab in recurrent or metastatic squamous cell carcinoma of the head and neck (R/M HNSCC), we conducted this real-world study to investigate the efficacy of pembrolizumab in patients with R/M HNSCC.

**Methods:**

This is a single-center retrospective study conducted in the Shanghai Ninth People’s Hospital Affiliated to Shanghai Jiao Tong University School of Medicine (Shanghai, China). Between December 2020 and December 2022, a total of 77 patients with R/M HNSCC were included into analysis. The primary endpoint of the study was overall survival (OS), and the secondary endpoints were progression-free survival (PFS), overall response rate (ORR)and toxicity.Efficacy was assessed according to RECIST version 1.1.SPSS 27.0 and GraphPad Prism 8.0 software were utilized to perform the statistical analysis.

**Results:**

By the cut-off date (February 28, 2023), the median OS,PFS and ORR were 15.97 months,8.53 months and 48.9% in patients treated with the pembrolizumab regimen in the first line therapy. Among these patients, 17 patients received pembrolizumab with cetuximab,and 18 received pembrolizumab with chemotherapy.We observed no significant differences between two groups neither in median OS (13.9 vs 19.4 months, P=0.3582) nor PFS (unreached vs 8.233 months, P= 0.2807). In the ≥2nd line therapy (n=30), the median OS, PFS and ORR were 5.7 months, 2.58 months and 20% respectively. Combined positive score (CPS) was eligible from 54 patients. For first line therapy, the median OS and PFS were 14.6 and 8.53 months in patients with CPS ≥1, and median OS and PFS were 14.6 and 12.33 months in patients with CPS ≥20. The immune-related adverse events (irAEs) were occurred in the 31 patients (31/77, 40.26%), and the most common potential irAEs were hypothyroidism (25.97%), and pneumonitis (7.79%).

**Conclusion:**

Our real-world results indicated that pembrolizumab regimen is a promising treatment in patients with R/M HNSCC

## 1 Introduction

China has a high incidence of head and neck squamous cell carcinoma (HNSCC). In 2021, there were 148,000 newly diagnosed cases of HNSCC, and regrettably, 78,000 patients succumbed to the disease (1). Squamous cell carcinoma in the oral cavity and oropharynx represents a significant portion of malignant head and neck tumors (2). The 5-year OS rate remained as low as 60%, indicating a generally poor clinical prognosis (3).

For patients with R/M HNSCC, systematic treatment and supportive therapy are the main choices, and the prognosis was poor (4, 5). Although the emergence of EXTREME regimen has improved the survival status of R/M HNSCC patients to some extent, the prognosis is still poor (6). In recent years, immune checkpoint inhibitors (ICIs) have been widely utilized in the treatment of R/M HNSCC.Over the past decade,the emergence of immunotherapy has promoted the treatment of R/M HNSCC. Pembrolizumab is an ICI that activates T cells’ immune surveillance of tumors by preventing programmed cell death-1(PD-1) from binding to programmed death-ligand 1(PD-L1) (7). The results of the KEYNOTE-040 and Checkmate 141 study revealed that the second-line treatment with ICI therapy could significantly prolong the OS of R/M HNSCC patients (8, 9). The subsequential KEYNOTE-048 study showed that patients with R/M HNSCC could obtain survival benefit from pembrolizumab regimen as the first line treatment (10). In addition, the combination of pembrolizumab and cetuximab also showed a favorite efficacy (11). Although these studies have achieved noticeable results,it is noteworthy that these studies have primarily concentrated on populations in Europe and the United States, with limited representation from Chinese and other Asian individuals. Given the potential variability in drug responses among different ethnic groups,it becomes essential to stratify patients based on their ethnic backgrounds.

To address these considerations, we conducted a single-center, real-world retrospective study to investigate the efficacy and safety of pembrolizumab in the treatment of Chinese patients with R/M HNSCC.

## 2 Methods

### 2.1 Patients and study design

This retrospective real-world study was conducted at a single center. It spanned from December 2020 to December 2022 and encompassed a total of 77 patients diagnosed with R/M HNSCC from the Shanghai Ninth People’s Hospital Affiliated to Shanghai Jiao Tong University School of Medicine (Shanghai, China). No age restrictions were imposed on the study participants.Inclusion criteria included that all patients were Chinese with squamous cell carcinoma of the oral cavity,oropharynx, hypopharynx,or larynx,and had recurrent or metastatic disease that was incurable with local therapies.ECOG PS 0-2. Patients with oropharyngeal cancer need to test p16 status. Lesion size was assessed via maxillofacial contrast-enhanced computed tomography (CT) scan. Eastern Cooperative Oncology Group Performance Status (ECOG PS) scores ranged from 0 to 2. Patients’ data were meticulously documented, encompassing information, such as age, gender, primary tumor location, human papillomavirus (HPV) status as indicated by p16 expression in oropharyngeal SCC patients, PD-L1 expression, type of tumor recurrence, prior treatments, including chemotherapy or cetuximab combination therapy,treatment commencement and completion dates, treatment-related adverse events,disease progression date, last follow-up date, and date of death. The study cohort consisted exclusively of Chinese patients, and the impact of the CPS on immunotherapy effectiveness was investigated. CPS was defined as the ratio of PD-L1-positive cells (comprising tumor cells, lymphocytes, and macrophages) to the total number of viable tumor cells, and it was categorized into three groups: CPS < 1, CPS ≥ 1, and CPS ≥ 20.

### 2.2 Imaging analysis

Imaging modalities consisted of oral contrast-enhanced CT and chest CT scans. To evaluate treatment response, CT scans were analyzed in adherence to the Response Evaluation Criteria in Solid Tumors (RECIST version 1.1).

### 2.3 Statistical analysis

SPSS 27.0(IBM,Armonk,NY,USA)and GraphPad Prism 8.0 (GraphPad Software Inc., San Diego, CA, USA) software were utilized to perform the statistical analysis. The primary endpoint of the study was OS (time from randomization to death from any cause), and the secondary endpoints were PFS (time from randomization to radiographically confirmed disease progression or death from any cause, whichever came first),ORR and toxicity. The ORR was defined as the percentage of patients achieving complete response (CR) or partial response (PR). Adverse events were graded according to the National Cancer Institute Common Terminology Criteria for Adverse Events (version 5.0). Patients were followed up until death or the last follow-up visit.PFS and OS and their 95% confidence interval (CI) were calculated by Kaplan-Meier method, and compared with log-rank test. Univariate and multivariate analysis were performed by COX regression analysis. Significant level <0.05 was defined as significant difference.

## 3 Results

### 3.1 Baseline demographic characteristics

Among 77 patients, 56 (72.7%) patients were male. The majority of patients presented with locally recurrent disease (57/77, 74.03%), and HPV-negative status (74/77, 96.10%). A total of 29 of all patients received pembrolizumab in combination with chemotherapy,25 patients received pembrolizumab plus cetuximab,6 patients received pembrolizumab in combination with nitolizumab,10 patients received pembrolizumab as monotherapy, 6 patients received pembrolizumab in combination with cetuximab and chemotherapy and 1 patient received pembrolizumab in combination with lenvatinib. Participants’ baseline characteristics are presented in **Table 1**. And in all study population, there were 42 patients (54.55%) over 60 years old. 65 patients (84.41%) had primary tumors in the oral cavity. We excluded tumors in other parts and metastatic tumors in the oral cavity or oropharyngeal. The pathological types of tumors in the patients who participated in the study were squamous cell carcinoma, but there was no limit to the degree of tumor differentiation. In a multivariate analysis of the factors affecting the OS (**Table 2**) and PFS (**Table 3**) of the enrolled patients,there was no significant difference in the characteristics of baseline patients, such as age, sex, tumor location, recurrence pattern and so on.

**Table 1:** Participants’ baseline characteristics.

Characteristics	Participants
Sex	
Female	21 (27.3%)
Male	56 (72.7%)
Age (years old)	
≥60	42 (54.5%)
<60	35 (45.5%)
Median Age	61
ECOG performance status	
0	0
1	70 (90.9%)
2	7 (9.1%)
Primary tumor site	
Oral	65 (84.4%)
Oropharyngeal	12 (15.6%)
Recurrence pattern	
Local or reginal recurrence only	57 (74.0%)
Local or reginal recurrence and distant metastases	12 (15.6%)
Distant metastases only	8 (10.4%)
HPV infection	
Positive	3 (3.9%)
Negative	74 (96.1%)
Previous lines of systemic therapy for recurrent or metastatic disease	
None	
pembrolizumab with chemotherapy	18 (23.3%)
pembrolizumab with cetuximab	17 (22.1%)
pembrolizumab with nimotuzumab	3 (3.9%)
pembrolizumab monotherapy	5 (6.5%)
pembrolizumab with cetuximab and chemotherapy	3 (3.9%)
pembrolizumab with lenvatinib	1 (1.3%)
≥1	
pembrolizumab with chemotherapy	11 (14.3%)
pembrolizumab with cetuximab	8 (10.4%)
pembrolizumab monotherapy	5 (6.5%)
pembrolizumab with nimotuzumab	3 (3.9%)
pembrolizumab with cetuximab and chemotherapy	3 (3.9%)

**Table 2:** Multivariate analysis for OS of R/M HNSCC patients.

Factors	Sig.	Exp(B)	95.0%Cl for Exp(B)	
			Lower	Upper
Sex (Male vs Female)	.977	.989	.466	2.102
Age (≥60vs<60)	.796	1.097	.543	2.215
Primary tumor site (Oral vs Oropharyngeal)	.766	.816	.215	3.101
Recurrence pattern	.673	1.161	.581	2.319
HPV infection (Positive vs Negative)	.694	1.593	.157	16.160
ECOG PS(1 vs 2)	.307	1.596	.650	3.919
First-line therapy vs ≥2nd line therapy	.018	.380	.171	.845
irAEs vs No irAEs	.018	.297	.108	.813

**Table 3:** Multivariate analysis for PFS of R/M HNSCC patients.

Factors	Sig.	Exp(B)	95.0%Cl for Exp(B)	
			Lower	Upper
Sex (Male vs Female)	.616	.836	.416	1.681
Age (≥60vs<60)	.684	1.152	.584	2.273
Primary tumor site (Oral vs Oropharyngeal)	.615	1.411	.369	5.392
Recurrence pattern	.142	.616	.323	1.175
HPV infection (Positive vs Negative)	.743	1.488	.138	15.991
ECOG PS (1 vs 2)	.061	2.386	.960	5.935
First-line therapy vs ≥2nd line therapy	.001	.265	.118	.592
irAEs vs No irAEs	.016	.297	.110	.800

### 3.2 Therapeutic effects

**Figure 1:**
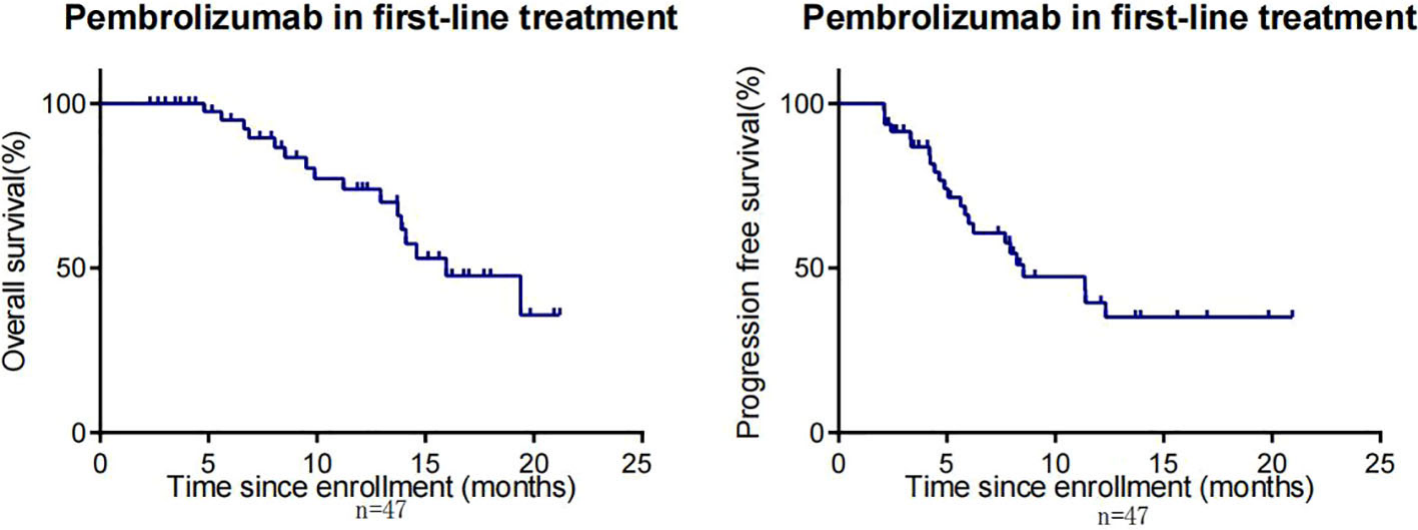
OS and PFS in R/M HNSCC patients who received first-line treatment with pembrolizumab.

**Figure 2:**
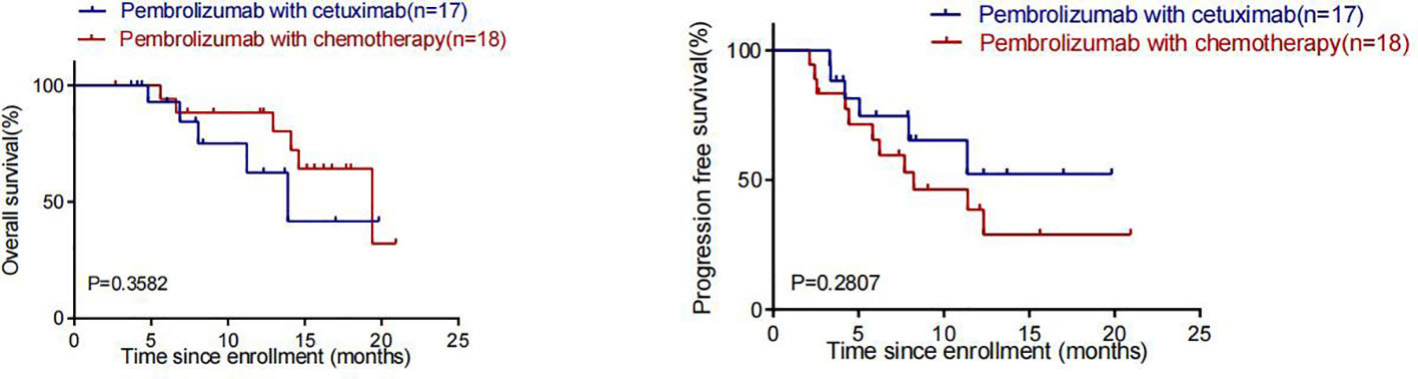
OS and PFS in patients who received pembrolizumab in Combination with cetuximab and pembrolizumab in combination with chemotherapy in first-line therapy.

**Figure 3:**
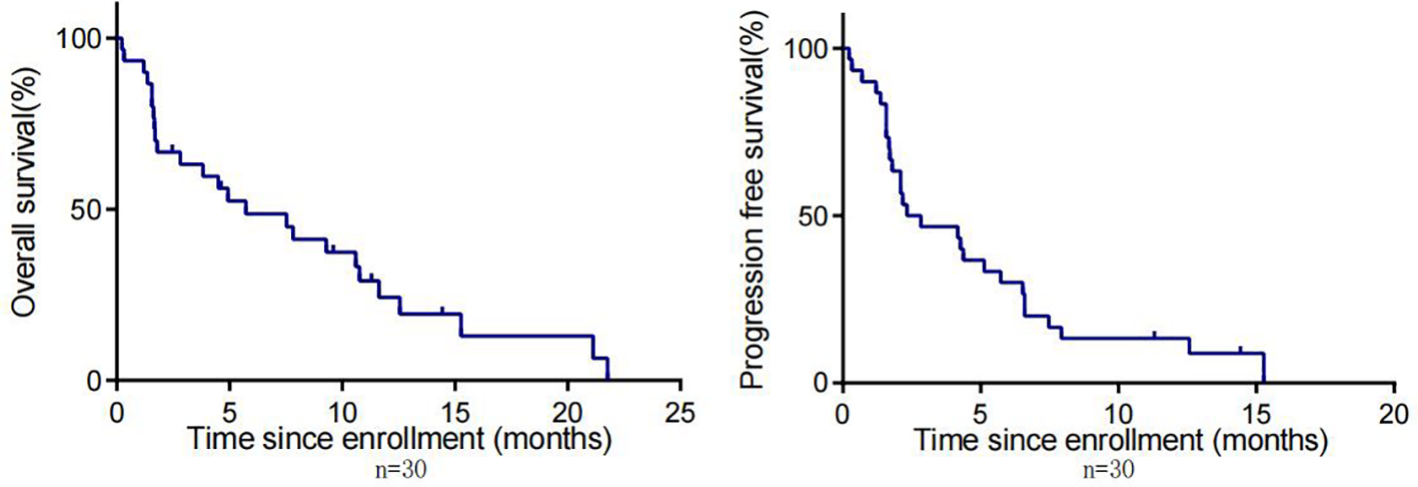
OS and PFS in patients with R/M HNSCC who received pembrolizumab in non-first-line therapy.

**Figure 4:**
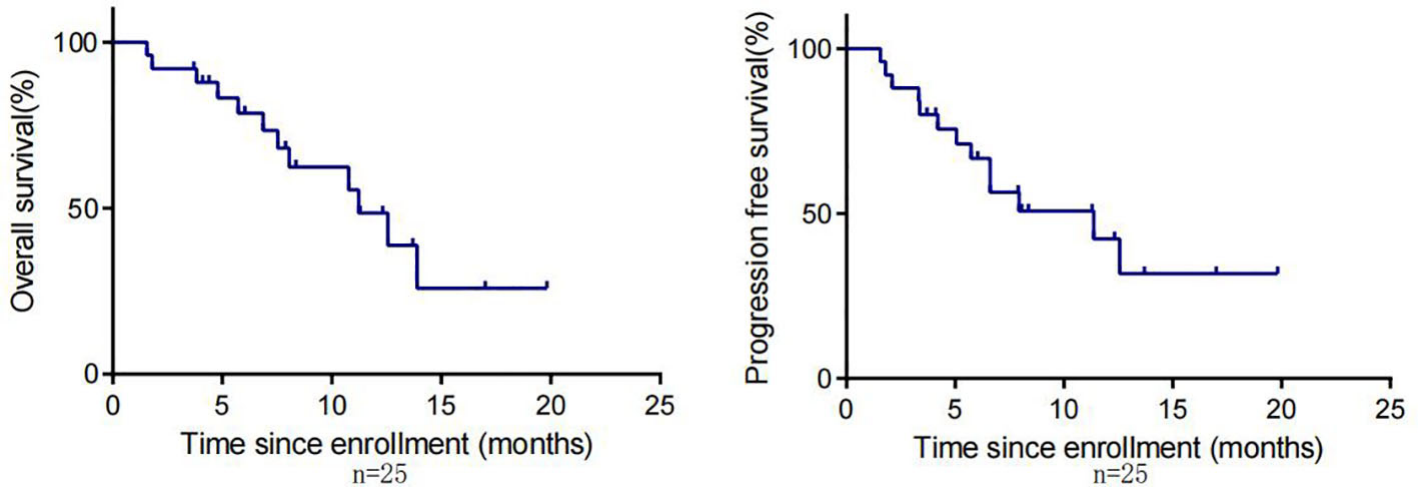
OS and PFS in R/M HNSCC patients treated with pembrolizumab in combination with cetuximab.

**Figure 5:**
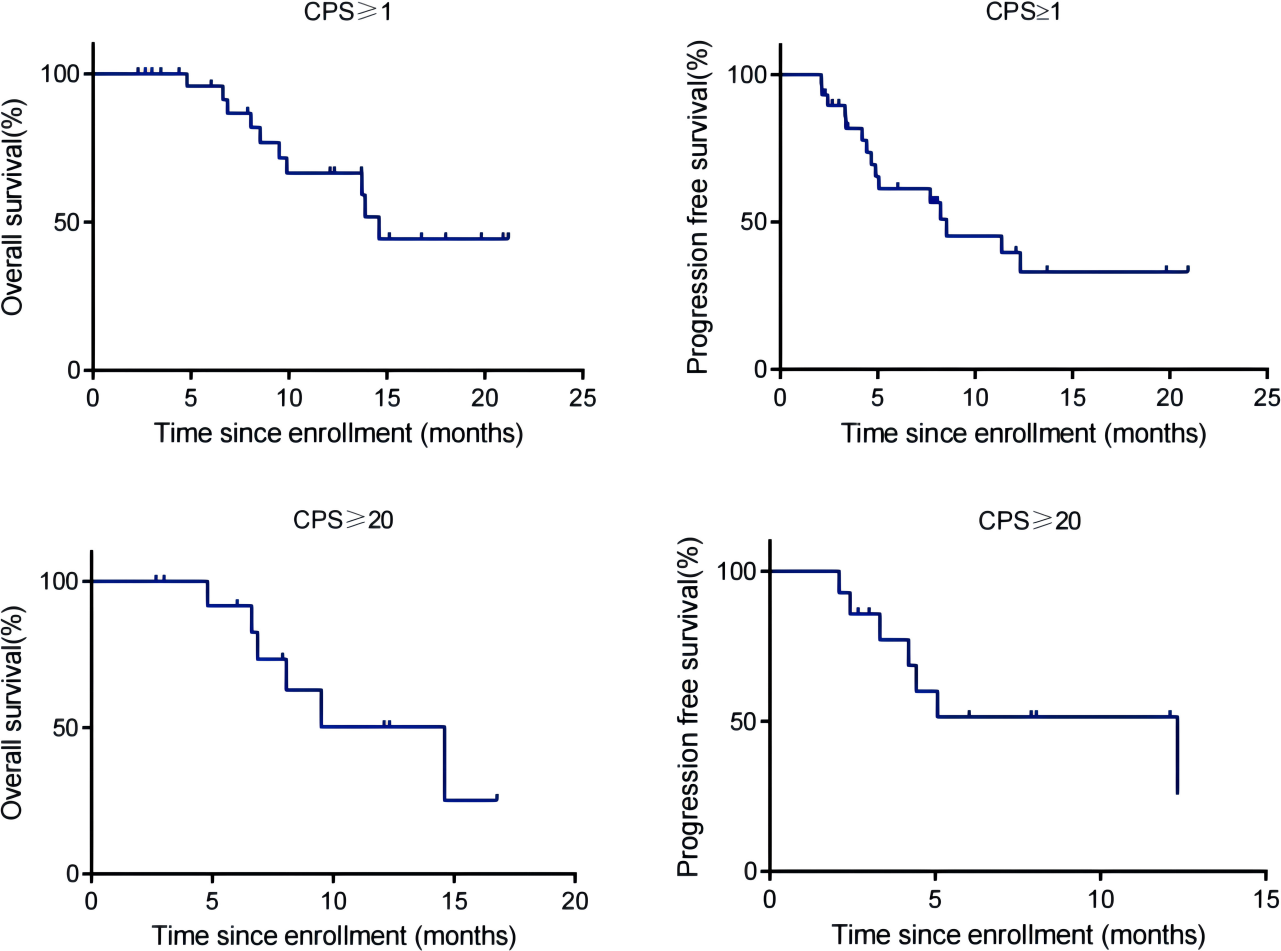
OS and PFS of R/M HNSCC patients with CPS ≥1 and CPS ≥20 treated with pembrolizumab as first-line therapy.

By the cut-off date (February 28, 2023), 41 patients had died. Among all patients who received treatment, 23 patients achieved PR and 3 patients achieved CR.The efficacy of some patients is shown below (**Supplementary 1**). Regarding patients who were treated with the pembrolizumab regimen in the first-line therapy (n=47), the median OS was 15.97 months,median PFS was 8.53 months (**Figure 1**), and ORR was 48.9%. Among 47 patients treated with the pembrolizumab regimen in the first-line therapy, 17 patients received pembrolizumab plus cetuximab,and 18 patients underwent pembrolizumab plus chemotherapy.No significant differences were found between two groups neither in the median OS (13.9 vs. 19.4 months, P=0.3582) nor PFS (unreached vs. 8.233 months, P=0.2807, **Figure 2**). In the ≥2nd line therapy (n=30), the median OS was 5.7 months, the median PFS was 2.58 months (**Figure 3**), and the ORR was 20%.Among patients who received pembrolizumab plus cetuximab (n=25), the median OS was 11.23 months,and the median PFS was 11.37 months (**Figure 4**). At the same time, we found that pembrolizumab plus cetuximab did not significantly prolong OS compared with pembrolizumab monotherapy either in the total population or in patients receiving first-line therapy. However, pembrolizumab plus cetuximab had a higher ORR in patients receiving first-line therapy(47.1%vs40%). And there was no more than level 3 irAEs in both groups. Out of all the patients analyzed, CPS data were available for 53 patients. Contemporary research identified that anti-PD1 immunotherapy can be potentiated in cases with the high PD-L1 expression in the tumor microenvironment,encompassing specific malignancies,such as HNSCC. Therefore,it was attempted to conduct subgroup analysis of PD-L1 expression status in patients receiving first-line treatment with pembrolizumab (12, 13). In patients with CPS ≥1 in the first-line therapy (n=29), the median OS was 14.6 months,and the median PFS was 8.53 months (**Figure 5**). In patients with CPS ≥20 in the first-line therapy (n=14), the median OS was 14.6 months,and the median PFS was 12.33 months (**Figure 5**). A total of 9 patients received other ICIs therapy before receiving pembrolizumab immunotherapy. The median OS and PFS were 12.57 months and 6.23 months, respectively, but the ORR was only 22.2%.

### 3.3 Safety

Among the 18 patients who received pembrolizumab plus chemotherapy in the first-line treatment, the treatment-related adverse events(TRAEs) were mainly rash,gastrointestinal reactions and fatigue, and 4 patients(4/18,22.22%)were suspended or discontinued due to TRAEs. However, among the 17 patients treated with pembrolizumab plus cetuximab,the TRAEs were mainly hypothyroidism and pneumonitis,and no TRAEs affected the treatment process. Based on the above results, it is suggested that compared with pembrolizumab combined with chemotherapy, the combination of pembrolizumab and cetuximab not only has comparable efficacy but is also safer.

The potential irAEs occurred in 31 (31/77, 40.26%) patients, and the most common potential irAEs were hypothyroidism (25.97%) and pneumonitis (7.79%). (**Supplementary 2**) Of these patients, three experienced grade 3 or higher irAEs, including hypothyroidism in two patients and grade 3 rash in another. All three patients suspended immunotherapy after the onset of irAEs,two patients with hypothyroidism improved after oral administration of thyroid hormones,and one of them continued to receive pembrolizumab immunotherapy. Another patient who developed a rash improved with oral and topical glucocorticoids and was subsequently maintained with cetuximab monotherapy.None of the three patients was hospitalized. At present,the exact mechanism of irAE has not been fully elucidated, but current studies have shown that irAE may be related to the bystander effect activated by T cells and is consistent with the mechanism of immune checkpoint inhibitors (14, 15). An observational study of non-small cell lung cancer in 270 patients with metastatic non-small cell lung cancer who received at least one treatment with anti-PD-L1 or anti-PD-1 antibodies showed that the efficacy of anti-PD-1 and anti-PD-L1 antibodies was associated with the pathogenesis of irAE.In this study, 89.3% of the patients received anti-PD-1 antibody treatment and the rest received anti-PD-L1 antibody treatment. IrAE occurred in 44 of all patients and had longer OS (not reached vs 8.21 months [hazard ratio (HR) 0.29;95% CI 0.18–0.46;p=0.001)] and PFS [5.2vs1.97months (HR0.42;95%CI0.32-0.57;p < 0.001)] than patients without irAE. According to irAE classification,there was no significant difference in OS,PFS,ORR and disease control rate(DCR) (16). In another retrospective study of non-small cell lung cancer, 43.6% of the 195 patients who received nivolumab developed irAE,and OS,PFS and ORR improved significantly compared with those without irAE (17). In an analysis of 114 patients with R/M HNSCC who received anti-PD-1 antibody treatment, ORR (30.6% vs 12.3%, p=0.02), PFS (6.9 vs 2.1months, p=0.0004)and OS (12.5 vs 6.8months, p=0.0007) in patients with irAEs were improved compared with those without irAEs. In multivariate analysis, the incidence of irAE was independently correlated with improved ORR (p=0.03), PFS (p=0.0009) and OS (p=0.003) (18).

**Figure 6:**
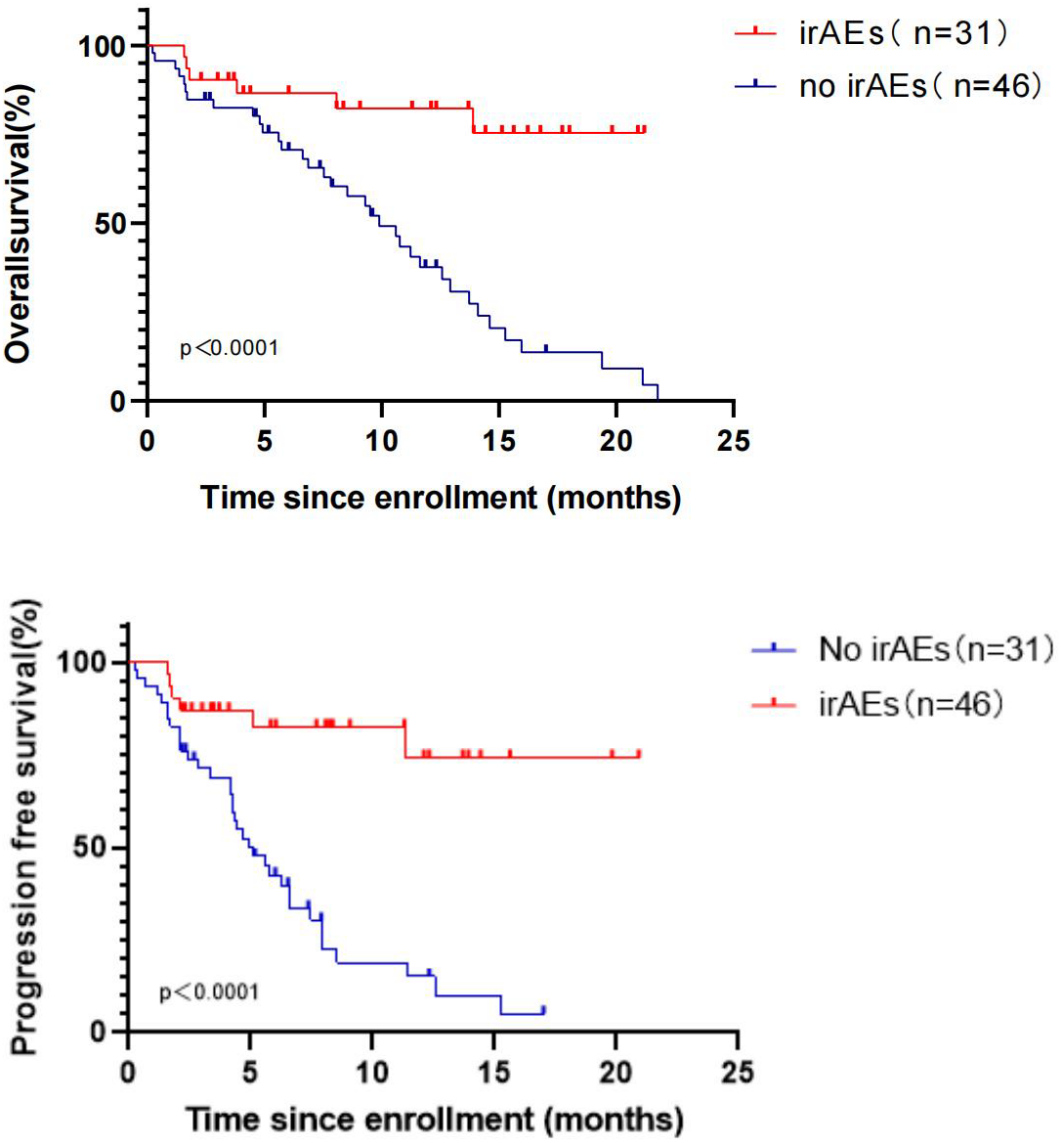
OS and PFS in R/M HNSCC patients with and without irAEs during treatment with pembrolizumab.

Therefore,the correlation between the occurrence of irAEs and treatment efficacy in R/M HNSCC patients undergoing pembrolizumab therapy was examined, yielding notable findings.Upon analyzing data from participants with and without irAEs, significant differences were identified in PFS between the two cohorts (unreached vs. 4.90 months, P < 0.0001). Importantly,6 out of 31 patients with irAEs had succumbed (6/31, 19.35%), in contrast to 35 out of 46 patients without irAEs (35/46, 76.09%), yielding a HR of 0.21 with a 95% confidence interval (CI) of 0.11-0.39. Furthermore, significant differences were found in OS between the two groups (unreached vs. 9.90 months, P < 0.0001) (**Figure 6**). In multivariate analysis, the incidence of irAE was independently correlated with improved PFS (p=0.016) (**Table 3**) and OS (p=0.018) (**Table 2**).

## 4 Discussion

Based on previous studies on the efficacy and safety of pembrolizumab, this single-center retrospective study was conducted to explore the efficacy and safety of pembrolizumab in the treatment of Chinese patients with R/M HNSCC in the real world. The results revealed that the median OS of patients treated with pembrolizumab in the first-line treatment was 15.97 months, the median PFS was 8.53 months, and the ORR was 48.9%. In patients with CPS ≥ 1, the median OS was 14.6 months, and the median PFS was 8.53 months. In patients with CPS ≥ 20, the median OS was 14.6 months, and the median OS was 12.33 month. These findings are basically consistent with the data reported in the KEYNOTE-048 study, demonstrating the satisfactory efficacy of pembrolizumab. However, the median OS and the median PFS of patients with non-first-line use of pembrolizumab were only 5.70 and 2.58 months respectively. The median OS of all patients treated with pembrolizumab plus cetuximab was 11.23 months. There were 17 patients who received the two-drug combination regimen in the first-line treatment, in which the median OS was 13.90 months, which was somewhat different from the median OS of 18.4 months for the two-drug combination regimen reported in previous studies (11). These differences could be attributed to the following factors:Racial disparities:Previous studies predominantly featured Caucasian participants,whereas our study exclusively involved Chinese subjects. Variations in genetic backgrounds and responses to treatment between racial groups can contribute to divergent outcomes. Study design variations: Previous investigations enforced stringent criteria, requiring participants to have no prior exposure to any immunotherapy or EGFR receptor inhibitors. In contrast, the present study is a real-world retrospective analysis, encompassing a more intricate spectrum of medications and regimens. Several patients had previously received different EGFR receptor inhibitors or alternative immune checkpoint inhibitors, potentially impacting the research outcomes. ECOG PS criteria: Prior studies concentrated on patients with ECOG PS scores of 0-1 as their inclusion criteria. In contrast, the present study included patients with ECOG PS scores of 2. Notably, a Japanese study has suggested that ECOG PS scores may influence pembrolizumab’s therapeutic efficacy (19). Therefore, the patient’s physical condition is an additional variable that might affect the results of the present study. It is essential to emphasize that, intriguingly, in the present study, 22.08% of patients with distant metastases achieved longer OS, compared to 63.64% of patients in a prior study with distant metastases. While it was hypothesized that the aforementioned factors may contribute to the research variations, further investigations are warranted to fully comprehend the underlying mechanisms.

There were 18 patients treated with pembrolizumab plus chemotherapy in the first-line treatment, and the median OS was 19.4 months, which is longer than the 13.0 months reported previously by the KEYNOTE-048. Although the median OS was longer than that of the pembrolizumab plus group, the median OS (13.9 vs. 19.4 months, P=0.3582) and the median PFS (unreached vs. 8.233 months, P= 0.2807) in the two groups showed no statistically significant difference. Among 30 patients who received pembrolizumab as a second-line treatment, the median OS was only 5.7 months, which was also slightly shorter than the median OS (8.4 months) reported in the KEYNOTE-040 study. In the KEYNOTE-040 study, the proportion of patients with CPS ≥ 1 reached 79%, while in the present study, patients with CPS ≥ 1 accounted for only 56.7% (17/30, 56.7%). CPS may affect the efficacy of immunotherapy. Thus, it could be speculated that this could be one of the reasons for the discrepancy between our research results and KEYNOTE-040 research’s findings. Consequently, pembrolizumab may be more effective as a first-line treatment for patients with R/M HNSCC, and the present study also demonstrated that patients who received first-line treatment would have a longer survival time.

In the present study, 31 of 77 patients experienced irAEs (31/77, 40.26%). As reported previously, cutaneous side effects are the most common irAEs, affecting >30% of patients (20). In the present study, the most common irAEs were hypothyroidism (25.97%) and pneumonia (7.79%), which are similar to the adverse reactions reported in the KEYNOTE-048 study. Prior research demonstrated that irAEs may be associated with tumor response in patients treated with immune checkpoint blockade (21). Therefore, an exploration was conducted and it was indicated that the median OS among 31 patients who experienced irAEs during treatment was significantly extended compared with 46 patients who did not encounter irAEs (unreached vs. 9.90 months, P < 0.0001). Additionally, the median PFS of the two patient groups exhibited significant differences (unreached vs. 4.90 months, P < 0.0001). The results revealed that the OS and PFS of patients with irAEs during treatment were significantly longer than those of patients without irAEs, suggesting that immunotherapy of these patients may be accompanied by higher efficacy. It was attempted to explore the reasons for this result and it was found that 25 (25/31, 80.65%) patients were treated with pembrolizumab as the first-line treatment among patients with irAEs, while 22 (22/46,47.83%) patients without irAEs were treated with pembrolizumab as the first-line therapy.Among the 31 patients with irAEs, there were only 4 patients with CPS < 1 and 8 patients with CPS ≥ 20, suggesting that the occurrence of irAEs may be related to the positive expression of PD-L1.Three patients experienced grade three or higher irAEs and the median OS and PFS were both 12.33 months.The remaining 28 patients all experienced grade 1-2 irAEs, and their median OS and PFS were 9.83 months and 5.93 months, respectively. The severity of IrAEs seems to be related to better survival status.However, the results of the study may be biased due to the small number of patients(3/31, 9.7%) who developed grade 3 and higher irAEs.The investigation of whether patients who developed irAEs during immunotherapy experienced improved outcomes merits further research. While irAEs are relatively infrequent, there are cases where patients discontinue treatment due to these adverse events. The challenge of finding a balance between treatment efficacy and adverse reactions, as well as exploring preventive measures, remains a subject deserving of the future research.

However, the present study had several limitations. As this was a single-center retrospective study, 77 patients who have been treated with pembrolizumab at our center were selected. Firstly, the sample size was small. Secondly, as a retrospective study, the tumor location, the pathological type and differentiation of the tumor, the site of recurrence or metastasis, the patient’s physical condition before immunotherapy, and the diversity of previous treatment options might lead to bias in the results of this study.A comprehensive multicenter study is required to assess the efficacy of pembrolizumab in the treatment of Chinese patients with R/M HNSCC. In the present study, no significant differences were noted in terms of median OS and PFS between the pembrolizumab plus cetuximab group and the pembrolizumab plus chemotherapy group. However, the incidence and severity of complications were significantly lower in the pembrolizumab plus cetuximab group compared with those in the pembrolizumab plus chemotherapy group. This indicates that the combination of pembrolizumab and cetuximab may potentially serve as a primary treatment option for R/M HNSCC, especially for patients who are either unable to tolerate or averse to chemotherapy. The incorporation of immunotherapy and targeted therapy presents an efficacious and safe alternative for this particular patient population.

In summary, this real-world single-center retrospective study conducted in China showed that pembrolizumab has a promising therapeutic efficacy as a first-line treatment for patients with R/M HNSCC. Furthermore, its safety is comparable to the Keynote-048 study. It was also found that the occurrence of irAEs may have a positive impact on patients’ survival. In the future, there will be continued patient follow-up to confirm these conclusions and make the data of this real-world study more comprehensive.

The emergence of immunotherapy has completely changed the treatment model of R/M HNSCC, providing a new option for these patients. Although HNSCC is mainly characterized by a high tumor mutational burden, which is favorable for immunotherapy, HNSCC is an immune-desert tumor that can evade immune recognition, suppress immune system activation, etc (22). How to overcome immune escape and maximize the therapeutic effect of HNSCC still needs further research. In addition, the expression level of PD-L1 is directly associated with the effects of immunotherapy. Patients with higher CPS tend to have better prognosis with immunotherapy, while a recent study demonstrated that the expression level of PD-L1 could vary throughout the course of HNSCC (23). It may be necessary to re-evaluate the expression level of PD-L1 in patients with recurrent disease to indicate further treatment. Checkmate-358 and other studies have shown that immunotherapy may be highly appropriate for early treatment, and the first-line treatment results are significantly superior to the posterior line (24), which is consistent with our research results. Compared with the previous standard regimen for R/M HNSCC, immunotherapy significantly improved patients’ survival while reducing adverse reactions. However, in the clinical practice, it was also found that some patients with high CPS had poor prognosis, and some patients experience rapid tumor growth, known as hyperprogression, shortly after initiating immunotherapy. At present, commonly used clinical regimens include pembrolizumab monotherapy or its combination with molecular targeted therapy or chemotherapy. However, there is a wide array of immunotherapy drugs available, such as nivolumab (a PD-1 inhibitor), atezolizumab (a PD-L1 inhibitor), ipilimumab (a CTLA-4 inhibitor), the recently introduced cardonirimab, etc. The effectiveness of single immunotherapy drugs is limited, prompting the consideration of multiple immunotherapy strategies. Immunogenic cell death (ICD) has been a significant focus in prior research. Typically, apoptotic tumor cells are not recognized by the body’s immune system, lacking immunogenicity. However, following treatments, such as radiotherapy or specific drugs, deceased tumor cells can activate the immune system. This activation enhances the function of dendritic cells (DC) in recognizing and presenting antigens, ultimately empowering cytotoxic T cells to eliminate tumor cells. This process is referred to as ICD. Substances that induce ICD are known as immunogenic cell death inducers,which include oxaliplatin, doxorubicin, and others (25–27).Whether the combination of ICD inducers with immunotherapeutic drugs yields superior therapeutic outcomes is a subject deserving of further investigation in the future.

At present, pembrolizumab is mainly utilized in the treatment of R/M HNSCC, although squamous cell carcinoma accounts for more than 80% of malignant tumors of the head and neck. Further research is essential to indicate whether pembrolizumab can produce the same promising efficacy in the treatment of other types of cancer.

## 5 Conclusions

In this real-world retrospective study, the survival status of 77 patients with R/M HNSCC treated at the Ninth People’s Hospital of Shanghai Jiaotong University School of Medicine was thoroughly examined. The results confirmed the promising efficacy and safety of pembrolizumab in the context of Chinese patients. Additionally, the combination of pembrolizumab and cetuximab also demonstrated noticeable effectiveness, particularly for patients with R/M HNSCC.

## Data availability statement

The original contributions presented in the study are included in the article/**Supplementary Material**. Further inquiries can be directed to the corresponding authors.

## Ethics statement

The studies involving humans were approved by Ethics Committee of the Ninth People’s Hospital of Shanghai Jiaotong University School of Medicine. The studies were conducted in accordance with the local legislation and institutional requirements. The participants provided their written informed consent to participate in this study.

## Author contributions

ZF: Conceptualization, Data curation, Formal analysis, Methodology, Resources, Software, Validation, Visualization, Writing – original draft. HJ: Conceptualization, Data curation, Formal analysis, Investigation, Methodology, Project administration, Resources, Software, Supervision, Validation, Visualization, Writing – original draft, Writing – review & editing. YW: Conceptualization, Methodology, Resources, Validation, Visualization, Writing – review & editing. XM: Conceptualization, Software, Writing – review & editing. HS: Formal analysis, Software, Visualization, Writing – review & editing. YL: Formal analysis, Methodology, Software, Writing – review & editing. DW: Data curation, Formal analysis, Methodology, Software, Validation, Writing – review & editing. MR: Formal analysis, Writing – review & editing. RH: Data curation, Methodology, Resources, Writing – review & editing. XG: Data curation, Formal analysis, Writing – review & editing. MZ: Data curation, Formal analysis, Software, Writing – review & editing. YX: Formal analysis, Writing – review & editing. GR: Conceptualization, Data curation, Funding acquisition, Investigation, Methodology, Project administration, Resources, Supervision, Validation, Visualization, Writing – original draft, Writing – review & editing.
