# Supplementary material for: The clinical outcome of pembrolizumab for patients with recurrent or metastatic squamous cell carcinoma of the head and neck: a single center, real world study in China
Source: Front Oncol. 2024 Feb 19;14:1360657. doi: 10.3389/fonc.2024.1360657 (PMC10910039; doi:10.3389/fonc.2024.1360657)
Supplement: Supplementary file 1 [file DataSheet_1.doc]

Supplement 1: representative images of imaging and histopathology of relevant patients
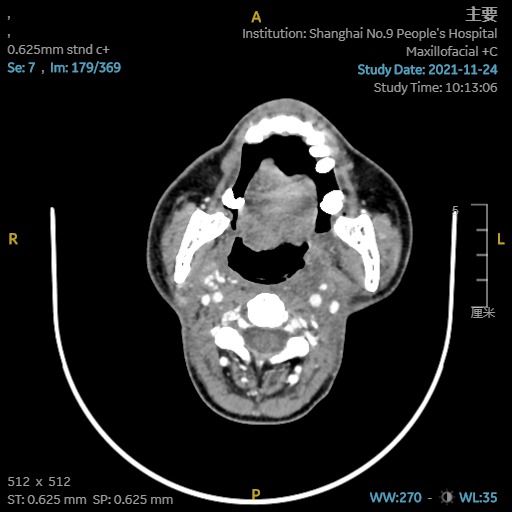

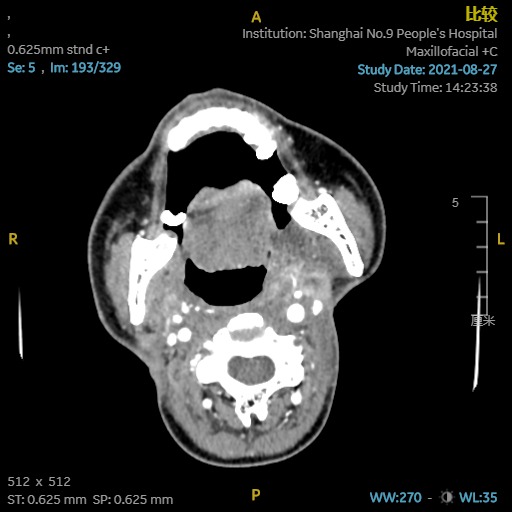


A B


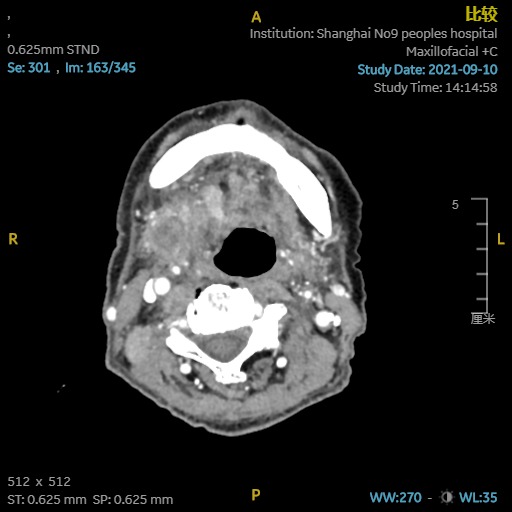

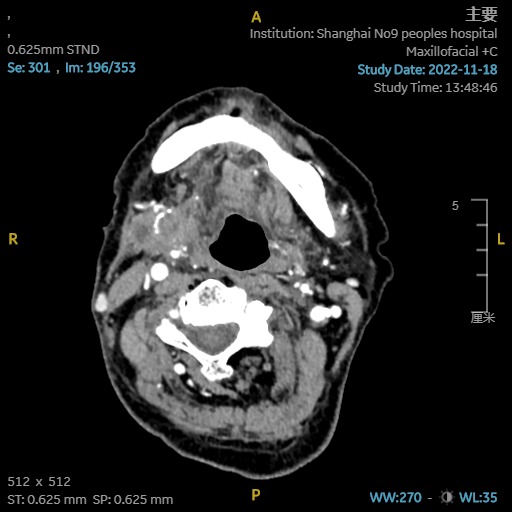


C D


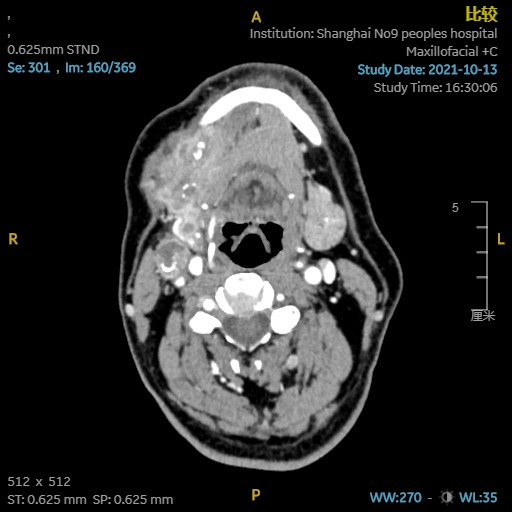

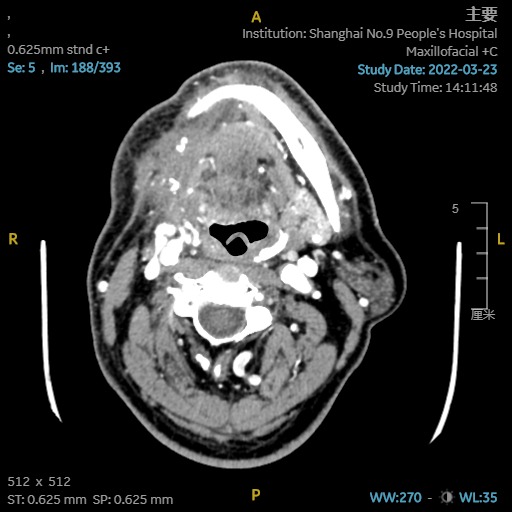


E F


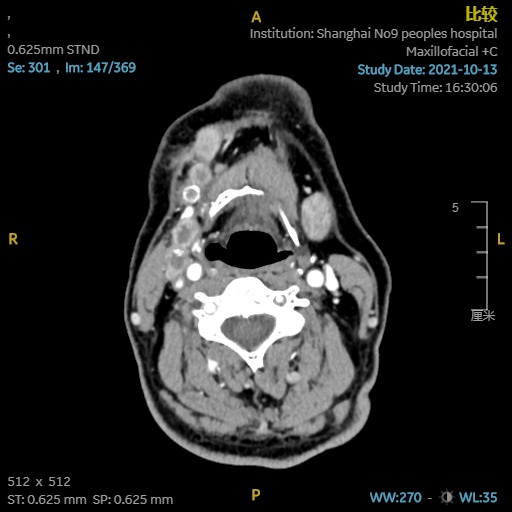

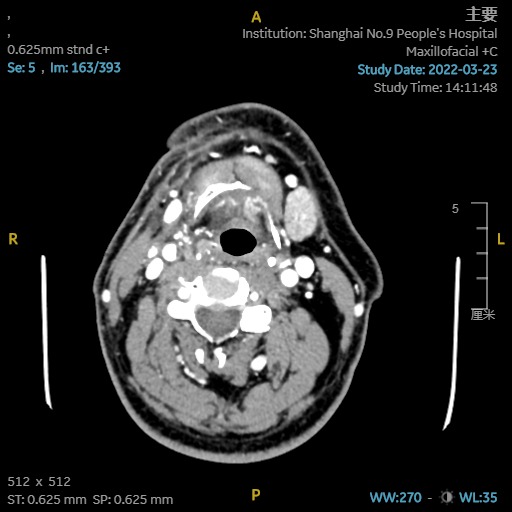


G H


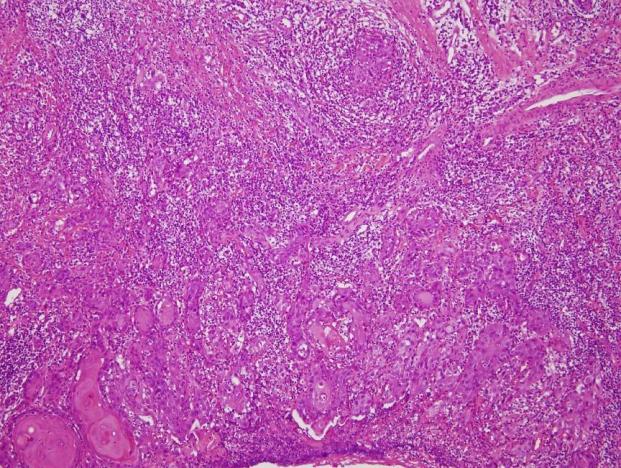

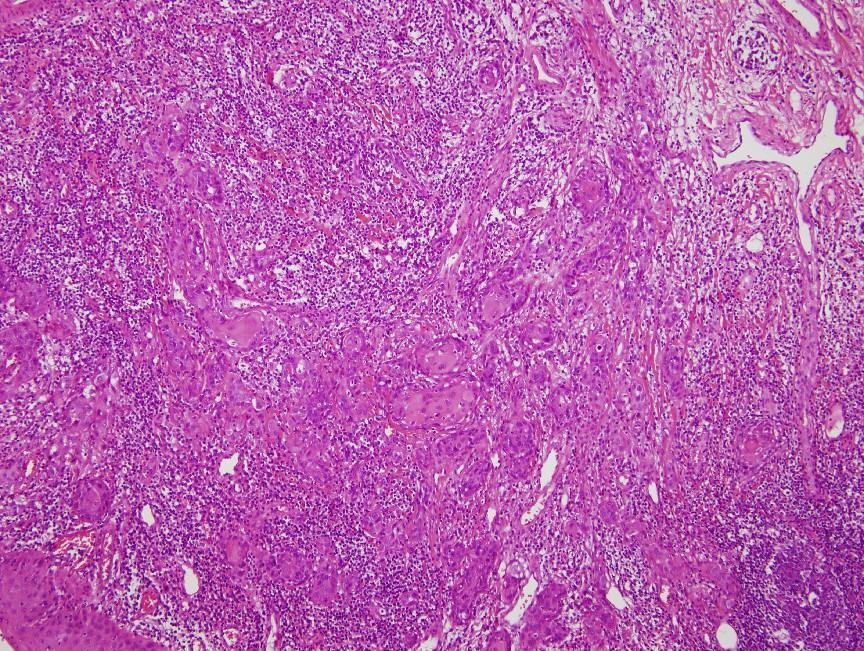


I J

A:SCC of the left oropharynx before treatment

B:SCC of the left oropharynx After four cycles of pembrolizumab combined with chemotherapy

C:SCC of right tongue before treatment

D:SCC of right tongue after ten cycles of pembrolizumab monotherapy

E,G:SCC of right mandibularwith with right cervical lymph node metastasis before treatment

F,H:SCC of right mandibularwith with right cervical lymph node metastasis after 5 cycles of pembrolizumab monotherapy

I,J:Major pathologic remission(MPR) after pembrolizumab immunotherapy

Supplement 2: immune-related adverse events for all patients(n=77)

|  | Grade 1-2 | Grade 3 and above |
| --- | --- | --- |
| Immune-related adverse events |  |  |
| Hypothyroidism | 18(23.4%) | 2(2.6%) |
| Rash acneiform | 10(13.0%) | 1(1.3%) |
| Nutritional disorders | 6(7.8%) | 0 |
| Pneumonia | 6(7.8%) | 0 |
| Fatigue | 2(2.6%) | 0 |
| Hand-foot syndrome | 1(1.3%) | 0 |
| Urinary tract Infection | 1(1.3%) | 0 |

The table records the occurrence of grade 1-2 and above irAEs. The data represent the number (%) of adverse events, and one patient may have multiple TRAEs at the same time. There were no deaths due to TRAEs.
